# Metabolic responses to pyruvate kinase deletion in lysine producing *Corynebacterium glutamicum*

**DOI:** 10.1186/1475-2859-7-8

**Published:** 2008-03-13

**Authors:** Judith Becker, Corinna Klopprogge, Christoph Wittmann

**Affiliations:** 1 Biotechnology Department, Institute for Biochemistry, Westfalian Wilhelms University Münster, Germany; 2 BASF AG, Research Fine Chemicals and Biotechnology, Ludwigshafen, Germany

## Abstract

**Background:**

Pyruvate kinase is an important element in flux control of the intermediate metabolism. It catalyzes the irreversible conversion of phosphoenolpyruvate into pyruvate and is under allosteric control. In *Corynebacterium glutamicum*, this enzyme was regarded as promising target for improved production of lysine, one of the major amino acids in animal nutrition. In pyruvate kinase deficient strains the required equimolar ratio of the two lysine precursors oxaloacetate and pyruvate can be achieved through concerted action of the phosphotransferase system (PTS) and phosphoenolpyruvate carboxylase (PEPC), whereby a reduced amount of carbon may be lost as CO_2 _due to reduced flux into the tricarboxylic acid (TCA) cycle. In previous studies, deletion of pyruvate kinase in lysine-producing *C. glutamicum*, however, did not yield a clear picture and the exact metabolic consequences are not fully understood.

**Results:**

In this work, deletion of the *pyk *gene, encoding pyruvate kinase, was carried out in the lysine-producing strain *C. glutamicum *lysC^fbr^, expressing a feedback resistant aspartokinase, to investigate the cellular response to deletion of this central glycolytic enzyme. *Pyk *deletion was achieved by allelic replacement, verified by PCR analysis and the lack of in vitro enzyme activity. The deletion mutant showed an overall growth behavior (specific growth rate, glucose uptake rate, biomass yield) which was very similar to that of the parent strain, but differed in slightly reduced lysine formation, increased formation of the overflow metabolites dihydroxyacetone and glycerol and in metabolic fluxes around the pyruvate node. The latter involved a flux shift from pyruvate carboxylase (PC) to PEPC, by which the cell maintained anaplerotic supply of the TCA cycle. This created a metabolic by-pass from PEP to pyruvate via malic enzyme demonstrating its contribution to metabolic flexibility of *C. glutamicum *on glucose.

**Conclusion:**

The metabolic flux analysis performed illustrates the high flexibility of the metabolic network of *C. glutamicum *to compensate for external perturbation. The organism could almost maintain its growth and production performance through a local redirection of the metabolic flux, thereby fulfilling all anabolic and catabolic needs. The formation of the undesired overflow metabolites dihydroxyacetone and glycerol, in the deletion mutant, however, indicates a limiting capacity of the metabolism down-stream of their common precursor glyceraldehyde 3-phosphate and opens possibilities for further strain engineering.

## Background

The biotechnological production of L-lysine by *Corynebacterium glutamicum *requires a continuous improvement of the lysine production process with a special focus on optimization of the production strains [1,2]. This includes the identification and implementation of genetic modifications that appear beneficial for production [3,4]. In previous work, pyruvate kinase was investigated as genetic target for improved production of lysine [5-7]. This enzyme, catalyzing the irreversible formation of pyruvate from phosphoenolpyruvate (PEP), is a key enzyme in the central pathways of energy production [8]. It is a target for the regulation by metabolites and plays a major role in the rate of energy synthesis, growth and lysine production [5,9]. Since pyruvate kinase catalyzes significant flux in *C. glutamicum *[10], its deletion is supposed to reduce the flux into the TCA cycle and the extent of carbon loss via CO_2 _formation. Moreover, pyruvate kinase-deficient strains can supply the required equimolar ratio of the two lysine precursors oxaloacetate and pyruvate through concerted action of the PTS and PEPC [5]. The deletion of pyruvate kinase in lysine producing strains of *C. glutamicum*, however, did not yield a clear picture and the exact metabolic consequences are not well characterized. Whereas pyruvate kinase deletion resulted in increased lysine production for different strains of the close relative *Brevibacterium flavum *[6,11,12], and during the major production phase of a batch process with *C. glutamicum *[7], production of lysine was strongly reduced in a strain of *C. lactofermentum *[5].

The exact metabolic consequences of deletion of pyruvate kinase in lysine producing *C. glutamicum *are still not well understood and the topic of the present work. Since single-gene knockouts can be potentially compensated by metabolic flux rerouting through alternative pathways [13,14], we combined quantitative physiological studies with ^13^C metabolic flux analysis in order to gain a deeper insight into the complex metabolic responses. For this purpose, ^13^C tracer experiments were combined with GC-MS analysis and metabolic and isotopomer balancing for the flux calculation. The potential of such studies for exploration of the central metabolism of *C. glutamicum *is illustrated from previous studies comparing fluxes in different mutants [15-19], during different phases of a lysine production process [20] or on different carbon sources [21,22]. As compared to these previous studies an extended experimental and computational setup was developed and applied here. This included an enlarged metabolic network with separate pools for pyruvate and phosphoenolpyruvate, two parallel tracer studies with 99% [1-^13^C] and 50 % [^13^C_6_] glucose and a significantly extended labeling data set with consideration of additional GC-MS fragments. In addition to the previous studies, the extended approach allowed to completely resolve the fine structure of the network around the pyruvate node, which was of special interest in the present work.

## Results

### Strain construction and validation

Deletion of the *pyk *gene (1428 bp) was obtained by allelic replacement with a shortened DNA fragment containing only the two flanking regions, but not the coding sequence of the gene. The resulting excision of the whole nucleotide sequence of *pyk *was verified by PCR analysis. Using a site specific primer set of forward and reverse primer (Table 1), the obtained DNA fragment was shortened by about 1500 bp when genomic DNA of lysC^fbr ^Δpyk was used as template instead of genomic DNA of the reference strain *C. glutamicum *ATCC 13032.

**Table 1:** Site-specific forward (*pyk*-F) and reverse (*pyk*-R) primer sequences used for verification of deletion of the *pyk *gene encoding pyruvate kinase in *C. glutamicum *by PCR.

**Primer direction**	**Primer sequence**
Forward	*pyk*-F: 5'-GATCC TCGAG CTCTA CTGAG CTGGT TTAT -3'
Reverse	*pyk*-R: 5'-GATCG GATCC CGGTC AACAT AGAGC TCA -3'

### Pyruvate kinase activity

*C. glutamicum *lysC^fbr ^Δpyk revealed complete absence of *in vitro *pyruvate kinase activity (< 0.6 mU mg^-1^), whereas the parent strain *C. glutamicum *lysC^fbr ^showed a strong specific activity of 1099 mU mg^-1 ^for this enzyme. PCR analysis and *in vitro *measurement of enzyme activity in crude cell extracts ensured the deletion of the *pyk *gene and further demonstrated that there is no remaining pyruvate kinase like activity in *C. glutamicum *lysC^fbr ^Δpyk. In the deletion strain, direct conversion of PEP into pyruvate thus is restricted to glucose uptake by the PTS.

### Influence of pyruvate kinase deletion on growth and production characteristics

**Figure 1:**
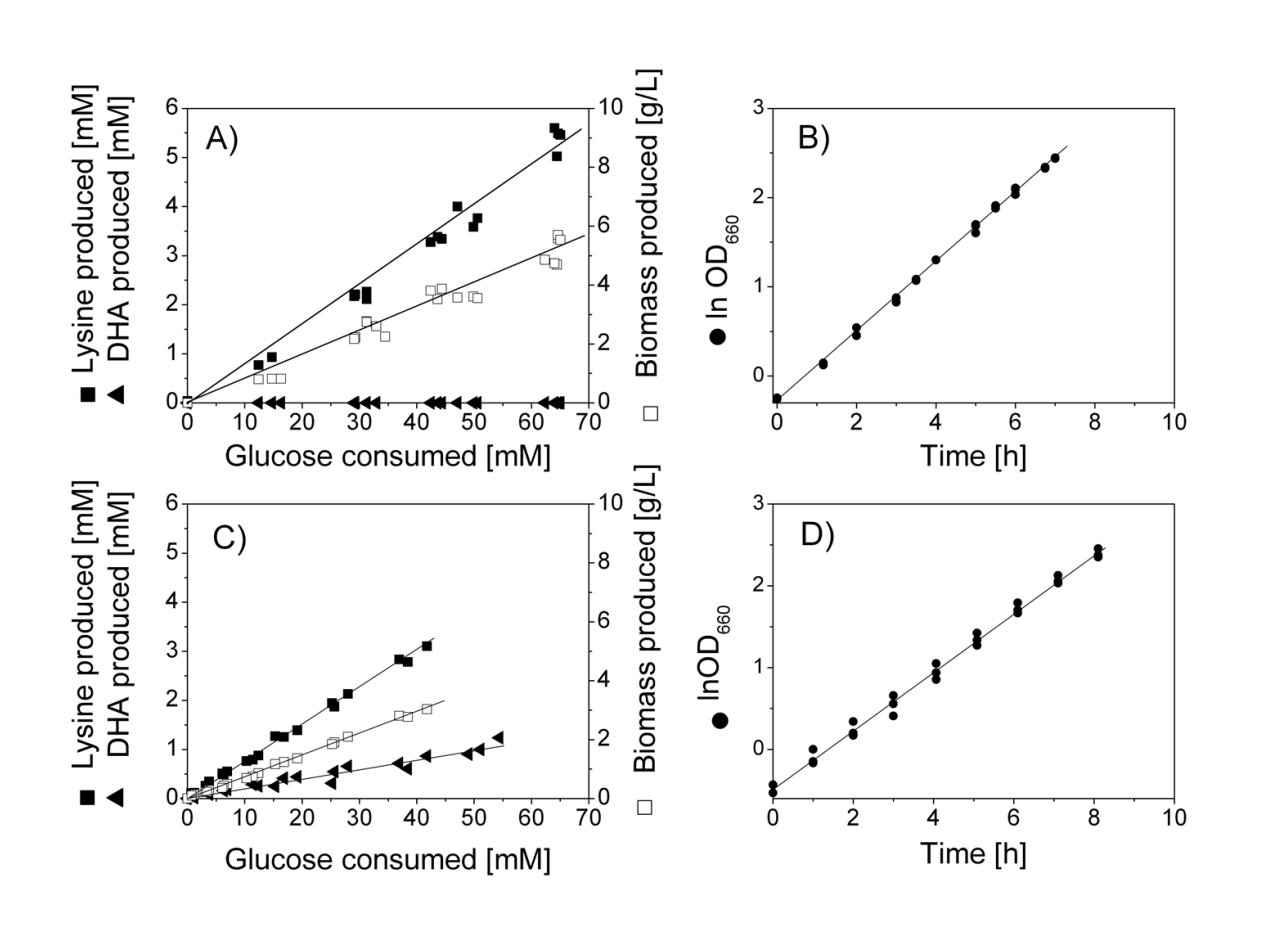
**Quantitative physiological characteristics of lysine producing *C. glutamicum *lysC^fbr ^(A, B) and lysC^fbr ^Δpyk (C, D) in batch culture on glucose.** The linear correlation between growth, production of lysine and dihydroxyacetone (DHA) and consumption of glucose indicates metabolic steady-state during the cultivation.

To investigate quantitative physiological effects of the pyruvate kinase deletion, lysine producing *C. glutamicum *lysC^fbr ^and its pyruvate kinase-deficient derivative *C. glutamicum *lysC^fbr ^Δpyk were grown in batch culture (Figure 1A–D). While both strains exhibited relatively similar growth characteristics, e.g. concerning specific growth rate, specific glucose uptake rate or biomass yield, yield and specific rate of lysine production appeared to be slightly lower in *C. glutamicum *lysC^fbr ^Δpyk (Table 2, 3). Moreover, lack of pyruvate kinase activity resulted in the formation of the overflow metabolites dihydroxyacetone (DHA) and glycerol whereas these compounds were not produced by the parent strain. Trehalose formation was slightly reduced. No significant difference was observed concerning the secretion of lactate which, however, was formed only in very low amount. Major kinetic and stoichiometric parameters, including specific growth rate or yield for biomass, lysine and by-products remained constant throughout the cultivation (Figure 1A – D). This clearly shows that both strains were in metabolic steady-state. In this context, the dissolved oxygen level was above 20 % throughout the whole cultivation so that sufficient oxygen supply for the cells was ensured. The pH remained constant in a range of 7.0 ± 0.2.

**Table 2:** Growth and production stoichiometry of lysine producing *C. glutamicum *ATCC 13032 lysC^fbr ^and lysC^fbr ^Δpyk during batch cultivation on glucose.

**Yield**	**lysC^fbr^**	**lysC^fbr ^Δpyk**
Y_X/Glc _[g/mol]	82.1 ± 1.5	77.1 ± 0.2

Y_Lys/Glc _[mmol/mol]	82.0 ± 2.9	73.9 ± 0.9
Y_DHA/Glc _[mmol/mol]	0.0 ± 0.0	20.8 ± 1.4
Y_Gly/Glc _[mmol/mol]	0.0 ± 0.0	6.2 ± 1.3
Y_Tre/Glc _[mmol/mol]	9.1 ± 0.4	2.9 ± 0.1
Y_Lac/Glc _[mmol/mol]	6.9 ± 0.6	7.5 ± 0.0

The data given are biomass yield (Y_X/S_), lysine yield (Y_Lys/S_) and yields on by-products glycerol (Y_Gly/S_), dihydroxyacetone (Y_DHA/S_), trehalose (Y_Tre/S_) and lactate (Y_Lac/S_). The yields were determined as slope of the linear fit between biomass or product formation, respectively, and substrate consumption (compare Figures 1A and 1C). The values given here represent the mean value from three parallel cultivation experiments with corresponding deviations.

**Table 3:** Growth and production kinetics of lysine producing *C. glutamicum *ATCC 13032 lysC^fbr ^and lysC^fbr ^Δpyk during batch cultivation on glucose.

**Rates**	**lysC^fbr^**	**lysC^fbr ^Δpyk**
μ [1/h]	0.38 ± 0.00	0.35 ± 0.00
q_Glc _[mmol/g/h]	4.60 ± 0.08	4.50 ± 0.01
q_Lys _[mmol/g/h]	0.38 ± 0.02	0.33 ± 0.00

The specific growth rate was estimated as slope from the semi logarithmic plot of cell concentration versus time (Figures 1B and D). The specific glucose uptake rate (q_Glc_) was calculated from the specific growth rate (μ) and the biomass yield (Table 2). The specific lysine production rate (q_Lys_) was calculated from q_Glc _and the lysine yield (Table 2). The values given here represent the mean value from three parallel cultivation experiments with corresponding deviations.

### Experimental design for metabolic flux analysis

**Figure 2:**
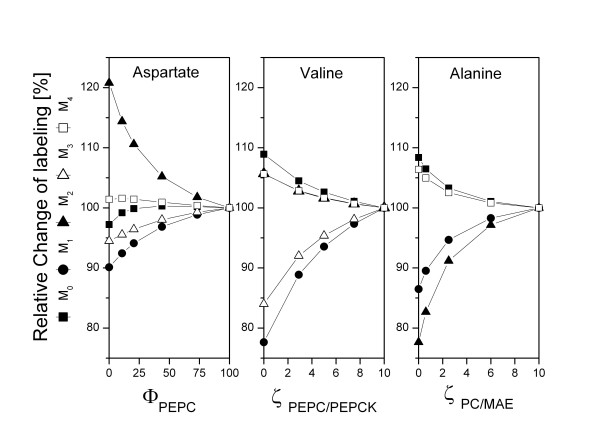
**Experimental design for quantification of flux parameters at the pyruvate node of *Corynebacterium glutamicum *with an equimolar mixture of [^13^C_6_] glucose and naturally labelled glucose**. Relative change of the mass isotopomer distribution of aspartate (m/z 418) with varied Φ_PEPC _(A), relative change of the mass isotopomer distribution of valine (m/z 288) with varied ζ_PEPC/PEPCK _(B), relative change of the mass isotopomer distribution of alanine (m/z 260) with varied ζ_PC/MAE _(C). The labelling patterns at sole contribution of PEPC (Φ_PEPC _= 100 %), and highly reversible fluxes at the pyruvate node (ζ_PEPC/PEPCK _= ζ_PC/MAE _= 10) are taken as reference point and set to 100 %. The flux parameters investigated here comprise Φ_PEPC _(flux partitioning between PEPC and PC), ζ_PEPC/PEPCK _(ratio of exchange flux to net flux between the pools of PEP and OAA/MAL catalyzed by PEPC and PEPCK) and ζ_PC/MAE _(ratio of exchange flux to net flux between the pools of PYR and OAA/MAL catalyzed by PC and MAE). The exact definitions for the flux parameters are given in the appendix. Unless varied the flux parameters reflect the situation for the parent strain *C. glutamicum *lysC^fbr ^(Figure 4).

In the present work, complete resolution of the fluxes through the different carboxylating and decarboxylating enzymes, i.e. PEPC, PC, phosphoenolpyruvate carboxykinase (PEPCK) and malic enzyme, was required, in order to study the exact influence of the pyruvate kinase deletion to these closely linked reactions. First, the metabolic network of *C. glutamicum *was extended by considering PEP and pyruvate as separate pools and PEPC, PC, PEPCK and malic enzyme as separate metabolic reactions. For this scenario, a previous approach utilizing labeling data from a single tracer study with [1-^13^C] glucose for *C. glutamicum *was significantly extended, since this is not capable to completely resolve all these fluxes, but provides lumped carboxylation and decarboxylation flux [17]. Concerning the central metabolic pathways, [1-^13^C] glucose is valuable for resolving the upper part of metabolism, in particular the oxidative PP pathway, glycolysis and the Entner-Doudoroff pathway, whereas the use of a mixture of [^13^C_6_] glucose and unlabeled glucose is particularly useful to resolve fluxes downstream of PEP, especially at the pyruvate node [23,24]. Due to this the experimental strategy was based on two parallel tracer studies with (i) [1-^13^C] glucose and (ii) a mixture of [^13^C_6_] glucose and unlabeled glucose to combine the information content available from the GC-MS labelling analysis of metabolites from the different tracer substrates for the flux calculation. Since it is known, that more detailed information for flux calculation can be obtained with GC-MS via additional analysis of fragment ions which contain only specific parts of the carbon skeleton of the analyte, we additionally considered a number of fragment ions from the proteinogenic amino acids which have previously proven useful for flux analysis in complex metabolic networks of prokaryotes [25] and eukaryotes [26]. Overall, 197 different mass isotopomer fractions were considered here for each strain, whereas the original simplified approach with only one single tracer experiment and less fragments measured considered only 29 mass isotopomer fractions. The extended approach was tested by computer based simulation studies to see, weather the additionally considered labeling information can be utilized to determine the additionally introduced free fluxes around the pyruvate node. For this purpose, sensitivities for the available mass isotopomer fractions and the flux parameters of interest were derived from partial derivatives as previously described [24]. The labeling patterns of a number of newly considered fragment ions were affected by variation of the free fluxes around the pyruvate node and thus contain sensitive information to determine these flux parameters of interest. This is exemplified for the study with [^13^C_6_] glucose and unlabeled glucose as tracer substrate and variation of the free flux parameters Φ_PEPC _(flux partitioning between PEPC and PC), ζ_PEPC/PEPCK _(exchange flux by PEPC and PEPCK) and ζ_PC/MAE _(exchange flux by PC and malic enzyme) which strongly influences the labelling pattern of analyzed metabolites (Figure 2). Using synthetic labelling data sets, a unique solution for the free fluxes was obtained in multiple parameter estimations with randomized varied starting values, demonstrating the observability and identifiability of all free fluxes in the network and the suitability of the developed extended approach.

### Tracer studies and flux parameter estimation

**Figure 3:**
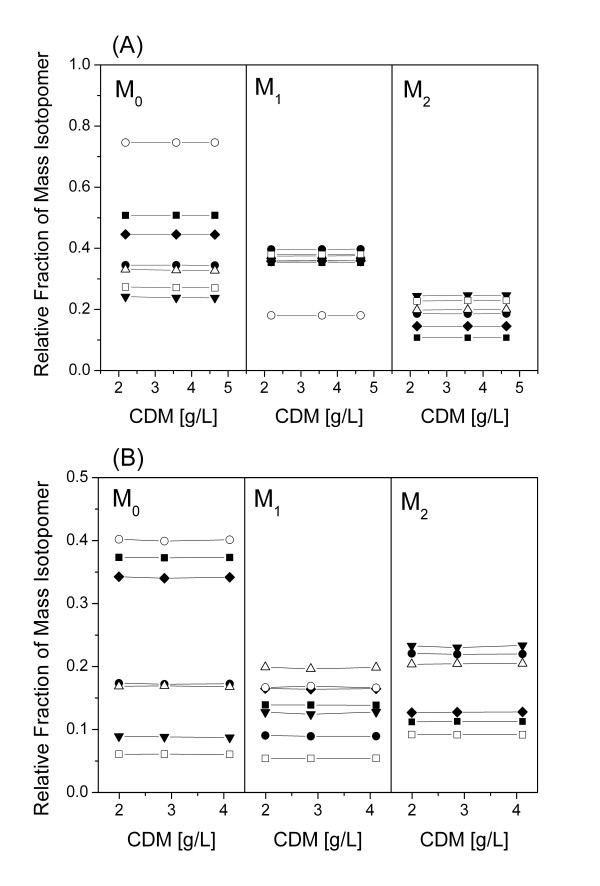
**Verification of isotopic steady-state during ^13^C tracer studies of *C. glutamicum *lysC^fbr ^Δpyk grown on [1-^13^C] glucose (A) and an equimolar mixture of [^13^C_6_] glucose and naturally labelled glucose (B).** The labelling patterns of the amino acids were determined from protein hydrolysates harvested at different cell dryx mass (CDM) concentrations during the cultivation. The amino acids shown here exemplarily stem form different parts of the metabolic network and comprise alanine (solid square), phenylalanine (open square), valine (closed circle), glycine (open circle), glutamate (closed triangle), threonine (open triangle) and serine (closed diamond). M_0 _(non labelled), M_1 _(single labelled) and M_2 _(double labelled) denote the relative fractions of the corresponding mass isotopomers.

**Figure 4:**
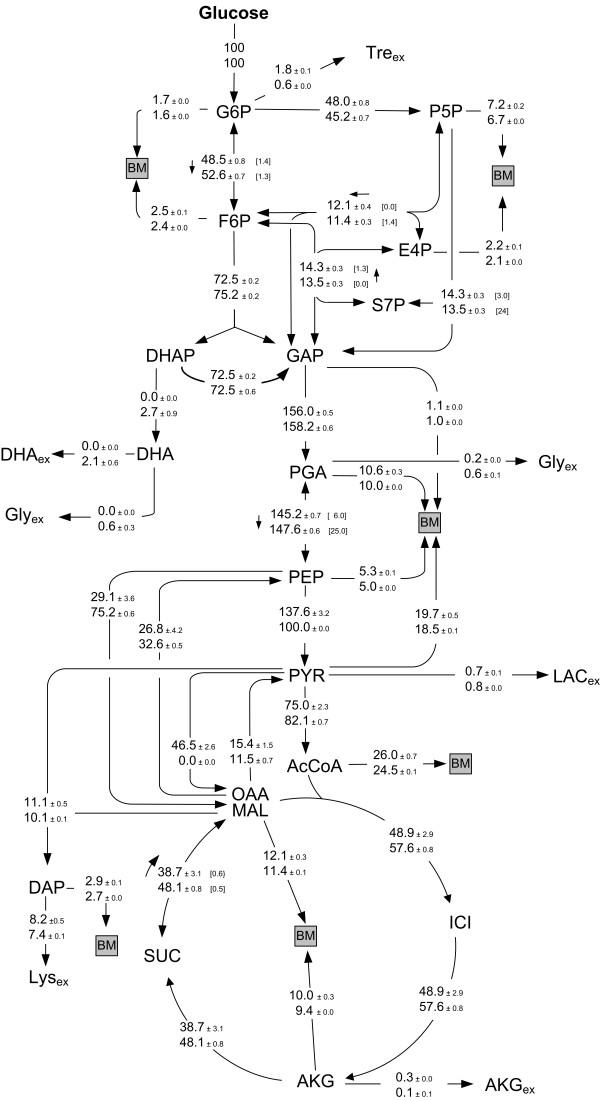
***In vivo *****carbon flux distribution in the central metabolism of lysine producing *C. glutamicum *lysC^fbr ^(top) and its pyruvate kinase deficient derivative *C. glutamicum *lysC^fbr ^Δpyk (bottom) during growth on glucose.** All fluxes are given as a molar percentage of the mean specific glucose uptake rate of q_Glc _= 4.6 mmol g^-1 ^h^-1 ^(for lysC^fbr^) and 4.5 mmol g^-1^h^-1 ^(for lysC^fbr ^Δpyk), which is set to 100 %. The errors reflect the corresponding 90 % confidence intervals for the different fluxes, obtained by Monte-Carlo analysis. For reversible metabolic reactions, the flux reversibility, i.e. the ratio of exchange flux to net flux, is additionally given in brackets and the direction of the net flux is indicated by an arrow.

Metabolic flux analysis, as applied here, requires a metabolic and isotopic steady-state of the investigated culture. The presence of metabolic steady-state was ensured from the constant growth and production behavior of the two strains (Figures 1A–D). Exemplified for different proteinogenic amino acids from the two parallel tracer studies sampled at different time points of the cultivation, also the ^13^C labeling patterns of the metabolites remained constant over time (Figure 3). This indicated isotopic steady-state during the cultivation, so that the flux distributions obtained can be taken as representative for each strain covering the whole cultivation period. The calculation of the metabolic fluxes was based on minimizing the deviation between the experimentally measured and the simulated mass isotopomer fractions. The approach comprised metabolic balancing during each step considering stoichiometric data on growth and product formation from three parallel cultivations (Table 2) and on anabolic demand for biomass precursors [27]. Obviously, an excellent fit was achieved (Table 4). The set of intracellular fluxes that gave minimum deviation between experimental and simulated labelling patterns was taken as best estimate for the intracellular flux distribution (Figure 4).

**Table 4:** Mass isotopomer fractions of amino acids from the cell protein and of secreted trehalose during cultivation of *C. glutamicum *lysC^fbr ^and lysC^fbr ^Δpyk on 99% [1-^13^C] glucose and on an equimolar mixture of 99% [^13^C_6_] glucose and naturally labelled glucose, respectively.

		lysC^fbr^				lysC^fbr ^Δpyk			
Analyte (*m/z*)		[1-^13^C] glucose		50% [^13^C_6_] glucose		[1-^13^C] glucose		50% [^13^C_6_] glucose	

		exp	calc	exp	calc	exp	calc	exp	calc
Ala_260_	M_0_	0.503	0.505	0.371	0.368	0.508	0.504	0.373	0.365
	M_1_	0.356	0.359	0.140	0.141	0.353	0.357	0.139	0.144
	M_2_	0.109	0.106	0.114	0.114	0.107	0.107	0.113	0.117
	M_3_			0.375	0.377			0.376	0.375

Ala_232_	M_0_	0.536	0.540	0.410	0.408	0.538	0.540	0.415	0.408
	M_1_	0.361	0.360	0.147	0.150	0.359	0.359	0.140	0.150
	M_2_			0.443	0.442			0.445	0.442

Val_288_	M_0_	0.341	0.339	0.171	0.163	0.344	0.339	0.173	0.161
	M_1_	0.396	0.401	0.091	0.091	0.397	0.400	0.089	0.092
	M_2_	0.189	0.188	0.218	0.218	0.186	0.188	0.220	0.219
	M_3_			0.236	0.236			0.236	0.236
	M_4_			0.107	0.111			0.106	0.113
	M_5_			0.177	0.181			0.177	0.180

Val_260_	M_0_	0.354	0.354	0.186	0.180	0.353	0.354	0.188	0.179
	M_1_	0.400	0.403	0.095	0.097	0.399	0.402	0.093	0.097
	M_2_	0.181	0.180	0.378	0.379	0.182	0.180	0.385	0.378
	M_3_			0.133	0.135			0.128	0.136
	M_4_			0.208	0.209			0.206	0.209

Val_186_	M_0_	0.388	0.393	0.200	0.195	0.387	0.393	0.205	0.194
	M_1_	0.411	0.418	0.088	0.091	0.408	0.417	0.084	0.091
	M_2_	0.154	0.154	0.395	0.397	0.155	0.155	0.404	0.396
	M_3_			0.116	0.114			0.109	0.115
	M_4_			0.201	0.204			0.198	0.204

Thr_404_	M_0_	0.333	0.331	0.173	0.167	0.327	0.329	0.167	0.161
	M_1_	0.375	0.377	0.199	0.196	0.376	0.377	0.199	0.195
	M_2_	0.197	0.196	0.195	0.200	0.200	0.198	0.205	0.209
	M_3_			0.227	0.232			0.224	0.228
	M_4_			0.206	0.205			0.206	0.207

Thr_376_	M_0_	0.372	0.371	0.198	0.198	0.366	0.370	0.194	0.192
	M_1_	0.378	0.380	0.251	0.249	0.380	0.380	0.256	0.252
	M_2_	0.184	0.183	0.298	0.297	0.187	0.184	0.296	0.296
	M_3_			0.253	0.256			0.254	0.260

Asp_418_	M_0_	0.328	0.331	0.171	0.167	0.324	0.328	0.167	0.161
	M_1_	0.374	0.376	0.197	0.196	0.374	0.376	0.200	0.195
	M_2_	0.200	0.197	0.195	0.200	0.202	0.198	0.205	0.209
	M_3_			0.228	0.232			0.223	0.229
	M_4_			0.209	0.205			0.205	0.207

Asp_390_	M_0_	0.370	0.371	0.200	0.198	0.364	0.369	0.195	0.192
	M_1_	0.376	0.379	0.250	0.248	0.379	0.379	0.256	0.252
	M_2_	0.185	0.184	0.294	0.297	0.188	0.184	0.293	0.296
	M_3_			0.255	0.257			0.256	0.260

Asp_316_	M_0_	0.405	0.408	0.214	0.212	0.398	0.406	0.209	0.206
	M_1_	0.382	0.387	0.252	0.251	0.384	0.387	0.259	0.255
	M_2_	0.160	0.158	0.290	0.293	0.164	0.159	0.288	0.291
	M_3_			0.244	0.244			0.244	0.248

Glu_432_	M_0_	0.241	0.245	0.090	0.087	0.238	0.244	0.087	0.084
	M_1_	0.358	0.366	0.127	0.124	0.359	0.365	0.128	0.125
	M_2_	0.245	0.242	0.230	0.233	0.246	0.242	0.233	0.231
	M_3_			0.238	0.241			0.245	0.244
	M_4_			0.183	0.185			0.180	0.185
	M_5_			0.132	0.130			0.127	0.132

Glu_330_	M_0_	0.335	0.331	0.156	0.147	0.334	0.330	0.155	0.144
	M_1_	0.396	0.398	0.140	0.141	0.393	0.397	0.141	0.146
	M_2_	0.193	0.194	0.336	0.336	0.195	0.196	0.340	0.332
	M_3_			0.178	0.185			0.179	0.190
	M_4_			0.189	0.191			0.185	0.189

Ser_390_	M_0_	0.443	0.443	0.344	0.345	0.445	0.442	0.342	0.342
	M_1_	0.361	0.362	0.162	0.163	0.359	0.361	0.165	0.165
	M_2_	0.145	0.145	0.123	0.123	0.145	0.146	0.128	0.127
	M_3_			0.371	0.369			0.366	0.366

Ser_362_	M_0_	0.478	0.480	0.383	0.386	0.478	0.480	0.382	0.385
	M_1_	0.375	0.375	0.169	0.170	0.375	0.374	0.174	0.171
	M_2_			0.448	0.445			0.444	0.444

Ser_288_	M_0_	0.519	0.521	0.407	0.407	0.520	0.521	0.405	0.406
	M_1_	0.368	0.368	0.151	0.149	0.368	0.368	0.154	0.150
	M_2_			0.443	0.444			0.440	0.443

Phe_336_	M_0_	0.277	0.271	0.060	0.057	0.271	0.268	0.060	0.057
	M_1_	0.382	0.386	0.054	0.052	0.380	0.383	0.054	0.052
	M_2_	0.225	0.230	0.093	0.091	0.230	0.231	0.092	0.091
	M_3_			0.144	0.137			0.142	0.137
	M_4_			0.144	0.141			0.142	0.141
	M_5_			0.142	0.141			0.140	0.142
	M_6_			0.143	0.145			0.144	0.146
	M_7_			0.102	0.107			0.103	0.107
	M_8_			0.059	0.063			0.061	0.063
	M_9_			0.060	0.066			0.061	0.065

Phe_234_	M_0_	0.311	0.313	0.072	0.069	0.306	0.309	0.073	0.069
	M_1_	0.405	0.409	0.057	0.055	0.403	0.408	0.057	0.056
	M_2_	0.209	0.208	0.157	0.157	0.212	0.210	0.157	0.156
	M_3_			0.129	0.123			0.129	0.124
	M_4_			0.176	0.175			0.173	0.175
	M_5_			0.126	0.124			0.128	0.125
	M_6_			0.153	0.160			0.154	0.159
	M_7_			0.062	0.065			0.063	0.065
	M_8_			0.068	0.073			0.067	0.072

Phe_302_	M_0_	0.712	0.711	0.402	0.389	0.709	0.708	0.395	0.388
	M_1_	0.209	0.210	0.182	0.178	0.210	0.212	0.184	0.181
	M_2_			0.416	0.432			0.422	0.432

Gly_246_	M_0_	0.747	0.743	0.403	0.399	0.746	0.738	0.401	0.398
	M_1_	0.180	0.184	0.164	0.164	0.180	0.188	0.166	0.167

Gly_218_	M_0_	0.822	0.821	0.432	0.436	0.822	0.818	0.433	0.435
	M_1_	0.178	0.179	0.467	0.463	0.178	0.182	0.467	0.463

Tyr_466_	M_0_	0.239	0.234	0.533	0.537	0.234	0.231	0.533	0.537
	M_1_	0.355	0.360	0.053	0.050	0.354	0.357	0.053	0.049
	M_2_	0.242	0.247	0.053	0.051	0.246	0.248	0.054	0.051
	M_3_			0.089	0.087			0.088	0.086
	M_4_			0.136	0.130			0.134	0.131
	M_5_			0.142	0.140			0.140	0.140
	M_6_			0.142	0.143			0.142	0.143
	M_7_			0.146	0.146			0.146	0.147
	M_8_			0.109	0.113			0.109	0.113

Tyr_302_	M_0_	0.716	0.711	0.067	0.071	0.714	0.708	0.069	0.072
	M_1_	0.206	0.210	0.062	0.068	0.208	0.212	0.064	0.068
	M_2_			0.397	0.389			0.395	0.388

Arg_442_	M_0_	0.199	0.195	0.050	0.049	0.187	0.193	0.048	0.046
	M_1_	0.332	0.341	0.105	0.106	0.328	0.340	0.118	0.105
	M_2_	0.257	0.267	0.183	0.183	0.265	0.268	0.174	0.180
	M_3_			0.233	0.232			0.217	0.232
	M_4_			0.207	0.204			0.199	0.206
	M_5_			0.146	0.149			0.157	0.151
	M_6_			0.075	0.078			0.087	0.080

Tre_361_	M_0_	0.060	0.061			0.062	0.063		
	M_1_	0.592	0.613			0.598	0.614		
	M_2_	0.214	0.204			0.210	0.202		

The data comprise experimental GC-MS data (exp) and values predicted by the solution of the mathematical model corresponding to the optimized set of fluxes (calc). The latter were corrected for the presence of natural isotopes [42, 43] to directly match the experimental data. M_0 _represents the amount of non-labelled mass isotopomer fraction, M_1 _the amount of singly-labelled mass isotopomer fraction and corresponding terms refer to a higher labelling.

### Metabolic flux response to deletion of pyruvate kinase

**Figure 5:**
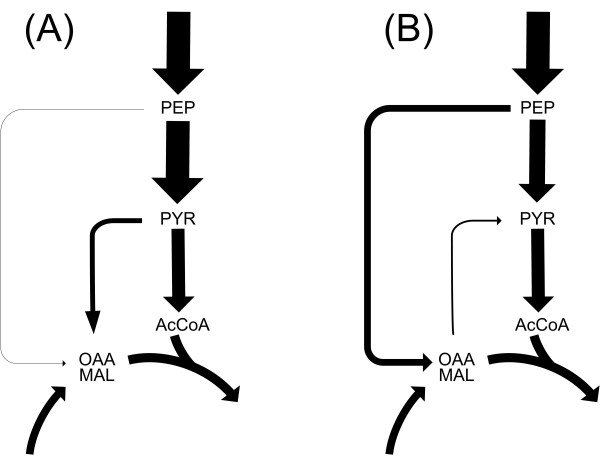
**Carbon net flux distribution at the pyruvate node of lysine producing *C. glutamicum *lysC^fbr ^(left) and its pyruvate kinase deficient derivative *C. glutamicum *lysC^fbr ^Δpyk (right) cultivated on glucose.** The actual flux values are represented by the thickness of the corresponding arrows.

The response of lysine producing *C. glutamicum *to pyruvate kinase deletion was now investigated on the level of metabolic carbon fluxes. It was interesting to see in detail, how *C. glutamicum *lysC^fbr ^Δpyk could compensate for the loss of this central glycolytic gene and almost maintain growth and production characteristics of the parent strain. As direct response to the genetic modification, the overall conversion flux from PEP into pyruvate decreased significantly from 138 % to 100 %. The metabolic flux distribution in Figure 4 reveals that the deletion of pyruvate kinase further resulted in local rerouting of the flux around the pyruvate node to by-pass the limited direct conversion from PEP into pyruvate. Responses in the pentose phosphate pathway and the TCA cycle were only rather weak. In detail, the two strains differed significantly in the relative contribution of PC and PEPC to anaplerotic supply of the TCA cycle. Whereas PC was the major anaplerotic enzyme in *C. glutamicum *lysC^fbr^, it was completely inactive in the deletion mutant which rather utilized PEPC for this purpose. The flux through the decarboxylating enzymes PEPCK and malic enzyme showed only slight differences between the two strains. Altogether pyruvate kinase deletion resulted in a strong shift of the anaplerotic net flux from pyruvate to PEP carboxylation and created a metabolic by-pass from PEP via oxaloacetate and malate towards pyruvate involving PEPC, malate dehydrogenase and malic enzyme (Figure 5). This by-pass enabled a sufficient supply of pyruvate so that a high flux through pyruvate dehydrogenase and TCA cycle was maintained. Overall, *C. glutamicum *lysC^fbr ^Δpyk could compensate for the gene deletion by local flux readjustment involving flexible utilization of its anaplerotic enzymes. The narrow confidence intervals underline that all carbon fluxes were estimated with very high precision (Figure 4). The flux differences discussed here are therefore clearly related to the deletion of pyruvate kinase. Since the specific glucose uptake rate was almost identical for the two strains (Table 3), all conclusions drawn for the relative fluxes also hold for the absolute flux values.

## Discussion

**Figure 6:**
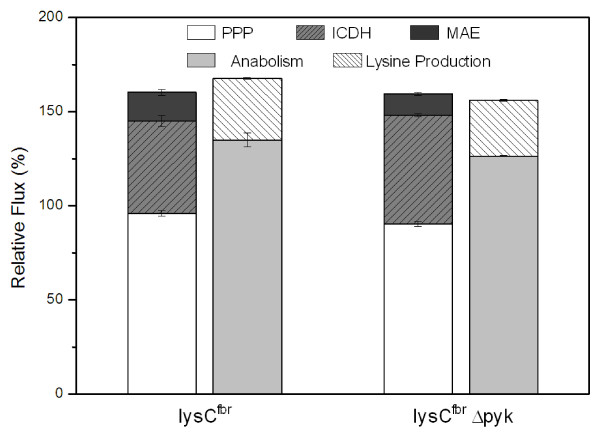
**NADPH balance for *C. glutamicum *lysC^fbr ^(left) and its pyruvate kinase deficient derivative *C. glutamicum *lysC^fbr ^Δpyk (right) considering glucose 6-phosphate dehydrogenase and 6-phosphogluconate dehydrogenase (PPP), isocitrate dehydrogenase (ICDH) and malic enzyme (MAE) as NADPH supplying reactions.** Anabolism with a stoichiometric demand of 16.4 mmol NAPDH (g cell dry weight)^-1 ^[27] and lysine production with a stoichiometric demand of 4 mol NADPH (mol lysine)^-1 ^were considered as NADPH consuming reactions.

In the present work the physiological responses of lysine producing *C. glutamicum *to deletion of the central glycolytic enzyme pyruvate kinase were studied. As shown, the pyruvate kinase deficient mutant showed slightly reduced lysine production, but could almost maintain the growth characteristics of the parent strain. The most striking consequence of pyruvate kinase deletion was a rearrangement of the fluxes through the anaplerotic reactions, i.e. a flux shift from PC to PEPC. This local flux rerouting in the deletion mutant created a metabolic by-pass via PEPC, malate dehydrogenase and malic enzyme. The conversion of oxaloacetate into malate as part of this by-pass is in the reverse direction of the net TCA cycle flux, rather forming oxaloacetate form malate, and would require a reversible inter conversion of these two metabolites. The metabolic network model applied here considers malate and oxaloacetate as a single pool, i.e. fully equilibrated labeling exchange between the two pools. The excellent fit of the labeling data thus supports the presence of a reversible inter conversion between malate and oxaloacetate. Further evidence comes from in vitro studies in *C. glutamicum *demonstrating the reversible inter conversion of malate and oxaloacetate by concerted action of the cytoplasmatic (MDH) and the membrane bound malate dehydrogenase (MDH) [28]. The activation of this by-pass is the key to compensate for the loss of pyruvate kinase activity and maintain the flux through the TCA cycle as well as through other central pathways including PPP, glycolysis or anabolism. The same metabolic by-pass is also activated in pyruvate kinase deficient *E. coli *during growth on glucose [13,29] and obviously displays a general strategy of microorganisms possessing both, PEPC and PC. In contrast, *B. subtilis *lacking PEPC cannot grow on glucose in the absence of pyruvate kinase [30,31]. Both strains exhibited significant flux through malic enzyme demonstrating the important role of this enzyme for the flexibility of the *C. glutamicum *metabolism. In the deletion mutant, malic enzyme is part of the metabolic by-pass created and thus contributes to the robustness of the metabolism against perturbation of the carbon flux. Its flux, i.e. its in vivo activity, in both strains shows that it also plays an important role for the flexibility of the cofactor metabolism in *C. glutamicum*. In the examined strains, lysine production is decoupled from feedback regulation thereby posing an increased demand for NADPH on the metabolism. As shown by the NADPH balance, malic enzyme is required to meet the cellular NADPH demand and contributes significantly to the supply of this cofactor (Figure 6). Previous studies already assumed such a physiological role e.g. during growth on fructose [32] or during lysine production [17]. The lower flux through malic enzyme as well as through the PPP in the pyruvate kinase deletion strain might be related to the increased flux through isocitrate dehydrogenase, resulting in enhanced NADPH supply via the TCA cycle. A co-regulation of NADPH supplying pathways, balancing the overall supply has been previously observed in lysine producing *C. glutamicum *[18].

The exact effect of deletion of pyruvate kinase on lysine production in *C. glutamicum *obviously depends on the production strain as well as on cultivation conditions. Whereas, upon deletion of pyruvate kinase, lysine production was enhanced in different strains of *B. flavum *[6,11], decreasing production was observed in *C. lactofermentum *[5] and, to some extent, also in the present work. Since the overall supply of NADPH did not significantly change, a possible explanation might be a limited availability of the lysine precursor oxaloacetate. The physiological characteristics and the intracellular carbon fluxes of *C. glutamicum *lysC^fbr ^and *C. glutamicum *lysC^fbr ^Δpyk, studied here, point at possible explanations. In this regard the accumulation of dihydroxyacetone and glycerol, specifically related to deletion of pyruvate kinase, appears interesting. These over flow metabolites are formed by *C. glutamicum*, when the flux entering into the lower glycolytic chain exceeds the capacity of reactions down-stream of glyceraldehyde 3-phosphate, e.g. during growth on fructose [21,32]. Under these conditions the bottleneck is attributed to glyceraldehyde 3-phosphate dehydrogenase and caused by an unfavorable ratio of NAD/NADH reducing the capacity of this enzyme. The glyceraldehyde 3-phosphate dehydrogenase flux, however, was not different between the two strains studied here, which does not support a contribution of this enzyme to the observed limitation. Other candidates appear more likely. As shown, the anaplerotic flux is completely shifted to PEPC in the deletion mutant. Compared to the parent strain, the overall flux through this enzyme is increased about 2.5 fold which could display the maximum capacity this enzyme can handle under these conditions. In this regard, strains of *C. glutamicum*, previously showing enhanced lysine production upon pyruvate kinase deletion, additionally contained a feedback-resistant variant of PEPC, insensitive to allosteric control by aspartate [11], whereas a double mutant, lacking pyruvate kinase and PEPC, exhibited seriously impaired glucose utilization [7]. It appears also possible that limited capacity of malic enzyme is involved in the observed limitation. Malic enzyme activity in the pyruvate kinase deficient wild type of *C. glutamicum *is not sufficient to enable growth on acetate or citrate, but growth can be restored by over expression of this enzyme [33].

## Conclusion

The present work is an evident example, that metabolic flux analysis is a powerful strategy for detailed quantitative investigation of production strains in order to obtain detailed understanding of the cellular response to genetic changes. The flux analysis performed illustrates the high flexibility of the metabolic network of *C. glutamicum *to compensate for external perturbation. The formation of the undesired overflow metabolites dihydroxyacetone and glycerol, in the deletion mutant, however, indicates a limiting capacity of the metabolism down-stream of their common precursor glyceraldehyde 3-phosphate and opens possibilities for further strain engineering. Based on the results of this study, over expression or de-regulation of PEPC in a pyruvate kinase negative production strain appear promising to overcome the formation of dihydroxyacetone and glycerol and enhance lysine production via increased supply of oxaloacetate.

## Methods

### Microorganisms

The reference strain *C. glutamicum *ATCC 13032 was derived from the American Type and Culture Collection (Manassas, USA). Deregulated lysine production through a feedback resistant aspartokinase was previously achieved by introduction of the point mutation T311I in the *lysC *gene (NCgl0247) leading to the lysine producing mutant *Corynebacterium glutamicum *lysC^fbr ^[16]. In the present work, pyruvate kinase was additionally deleted through allelic replacement of the *pyk *gene (NCgl2008), resulting in the pyruvate kinase deficient mutant *C. glutamicum *lysC^fbr ^Δpyk. The primer sequences used for verification of *pyk *deletion are given in Table 1.

### Media

Complex medium containing 5 g L^-1 ^glucose, 5 g L^-1 ^yeast extract, 10 g L^-1 ^tryptone, 5 g L^-1 ^NaCl and 18 g L^-1 ^agar was used for agar plates. First pre-cultures were grown in the same medium without agar. Second pre-cultivation and main cultivation was performed in minimal medium with 80 mM glucose as carbon source. The minimal medium additionally contained per liter: 0.055 g CaCl_2_· 2H_2_O, 0.2 g MgSO_4 _· 7H_2_O, 1.0 g NaCl, 16.0 g K_2_HPO_4_, 2.0 g KH_2_PO_4_, 5.0 g (NH_4_)_2_SO_4_, 0.5 mg biotin, 1 mg Ca-panthothenic acid, 1 mg thiamine · HCl, 20 mg FeSO_4_, 30 mg 3,4-dihydroxybenzoic acid and 10 ml of a 100 × trace element solution [34]. In tracer experiments for metabolic flux analysis, naturally labeled glucose was replaced by 99 % [1-^13^C] glucose or by an equimolar mixture of naturally labeled and 99 % [^13^C_6_] glucose.

### Cultivation

Single colonies from agar plates incubated for 24 h at 30°C were used to inoculate the first pre-culture which was grown for 8 h in 500 mL baffled shake flasks with 50 mL complex medium. Subsequently, cells were harvested by centrifugation (8,800 × g, 2 min, 4°C), washed with sterile 0.9 % NaCl, and used as inoculum for the second pre-culture (50 ml minimal medium in 500 mL baffled shake flasks). Physiological studies comprised three replicate cultivations for quantification of growth and production characteristics (500 ml baffled shake flasks with 50 mL minimal medium) and two parallel tracer cultivations with ^13^C labeled glucose (100 mL flasks with 10 mL medium) for each strain. All cultures were inoculated with exponentially growing cells from the second pre-culture and incubated at 30°C and 230 rpm on a rotary shaker (shaking diameter 5 cm, Multitron, Infors AG, Bottmingen, Switzerland). During cultivation, dissolved oxygen concentration was monitored via immobilized sensor spots on the flask bottom containing a fluorophor with oxygen dependent luminescent decay time [35].

### Chemicals

Yeast extract and tryptone were obtained from Difco Laboratories (Detroit, USA). Ninety-nine % [1-^13^C] glucose and 99 % [^13^C_6_] glucose were purchased from Campro Scientific (Veenendaal, The Netherlands). All other chemicals were purchased from Sigma, Merck (Darmstadt, Germany), or Fluka (Buchs, Switzerland) and were of analytical grade.

### Substrate and product analysis

Samples taken during the cultivation were analyzed for concentrations of biomass, substrates and products. Cell concentration was determined by measurement of the optical density at 660 nm (Novaspec II, Pharmacia Biotech, Little Chalfont, UK). If necessary, samples were diluted on an analytical balance (CP255D, Sartorius, Göttingen, Germany) to obtain absorbance values below 0.3. The correlation factor between cell dry mass (CDM) and optical density was determined as CDM = 0.382 × OD (gram per litre) for *C. glutamicum *lysC^fbr ^and 0.393 × OD (gram per litre) for *C. glutamicum *lysC^fbr ^Δpyk. For the analysis of extracellular substrates and products, cultivation supernatant was obtained by centrifugation of 1 mL broth (5 min, 16.000 × g, Biofuge Fresco, Heraeus Instruments, Hanau, Germany). Concentration of glucose, trehalose, glycerol, dihydroxyacetone (DHA) and organic acids was quantified in 1:10-diluted cultivation supernatant by HPLC (Kontron Instruments, Neufahrn, Germany). Separation was carried out on an Aminex HPX-87H column (300 × 7.8 mm; Bio-Rad, Hercules, USA) at 45°C with 5 mM H_2_SO_4 _as mobile phase and a flow rate of 0.5 ml min^-1^. Refraction index (sugars, glycerol) and UV absorption at 210 nm (organic acids, DHA) was used for detection. Amino acid quantification in cultivation supernatant was performed as described previously [36].

### Pyruvate kinase assay

Crude cell extract was prepared as described previously [37]. Cell debris was removed by centrifugation (9,800 × g, 2 × 30 min, 4°C) and the obtained supernatant was used for determination of pyruvate kinase activity and protein content by the method of Bradford [38]. Enzyme activity was determined as described by Netzer et al. [33]. The reaction was carried out in a total volume of 1 ml at pH 7.0 containing 100 mM Tris/HCl, 15 mM MgCl_2_, 1 mM ADP, 0.25 mM NADH, 5.5 U of lactate dehydrogenase, 10 mM PEP and 10 μl of the crude cell extract. Negative controls were carried out without PEP or without cell extract, respectively.

### Mass spectrometric labeling analysis

Mass isotopomer fractions of amino acids from hydrolyzed and lyophilized cell protein and of trehalose from lyophilized culture supernatant, harvested during the exponential phase from the tracer studies, were determined by GC-MS [19,39]. Sample preparation and measurement was performed as described previously [16]. The mean experimental error for the mass isotopomer fractions measured in triplicate was about 0.4 %.

### Metabolic network and biomass requirements

The metabolic network of *C. glutamicum *comprised all central metabolic pathways, i. e. glycolysis, PPP and TCA cycle [16]. In *C. glutamicum*, pyruvate carboxylase (PC), PEP carboxylase (PEPC), malic enzyme (MAE) and phosphoenolpyruvate carboxykinase (PEPCK) link glycolysis and TCA cycle through inter conversion of C_3 _and C_4 _metabolites [40]. In contrast to previous work, where carboxylation and decarboxylation, respectively, were only regarded as lumped fluxes all single enzymes were considered as separate reactions. For this purpose, PEP and pyruvate were considered as separate metabolic pools. Additionally the pathways for the biosynthesis of lysine and different by-products and the anabolic pathways from intermediary precursors into biomass were implemented. For glycine synthesis two possible routes were considered, i.e. via serine and via threonine aldolase [41]. Enzymatic steps regarded reversible were transaldolase and transketolase, phosphoglucose isomerase, the reactions of the lower glycolytic chain interconnecting PEP and 3-phosphoglycerate and of the TCA cycle interconnecting succinate and oxaloacetate. The precursor demand for biomass formation was taken from the biomass composition and the corresponding pathway stoichiometry of *C. glutamicum *[27]. For the pyruvate kinase deletion strain, the flux converting PEP into pyruvate was set equal to the glucose uptake flux. This was justified from the fact that no pyruvate kinase-like activity remained in this strain, so that the direct conversion of PEP into pyruvate was restricted to the glucose PTS.

### Estimation of metabolic flux

Metabolic flux distribution of *C. glutamicum *was determined by minimizing the deviation between the experimental and simulated GC-MS mass isotopomer fractions using a metabolic model implemented in Matlab 7.0 (Mathworks Inc., Nattick, USA) on a personal computer with isotopomer and metabolite balancing and the solver fmincon implemented in Matlab as previously described [18]. Hereby, the labelling data simulated by the isotopomer model were corrected for natural isotopes [42,43]. The parameter estimation involved two parallel metabolic networks for calculation of the ^13^C label distribution in order to consider the labelling data from both parallel tracer experiments in the same parameter optimization. The network was over determined. A least square approach was therefore possible. As error criterion a weighted sum of least squares (SLS) was used. For both strains, identical flux distributions were obtained with multiple randomized initialization values for the free flux parameters, suggesting that global minima were identified. Statistical analysis of the obtained fluxes was carried out by a Monte-Carlo approach [18]. From the obtained data 90 % confidence limits for the single parameters were calculated.

## Appendix

Flux partitioning ratio (Φ) and flux reversibility (ζ) were defined as relative flux into one of the two branches, and as ratio of backward or exchange flux to the net flux in the forward direction, respectively [18]. For the pyruvate node with the reactions catalyzed by phosphoenolpyruvate carboxylase (PEPC), pyruvate carboxylase (PC), phosphoenolpyruvate carboxykinase (PEPCK), and malic enzyme (MAE) the definitions were as follows:

$$\Phi_{\text{PEPC}}=\frac{\nu_{\text{PEPC}}}{\nu_{\text{PEPC}}+\nu_{\text{PC}}}$$

$$\zeta_{\text{PEPC/PECK}}=\frac{\nu_{\text{PEPCK}}}{\nu_{\text{PEPC}}-\nu_{\text{PEPCK}}}$$

$$\zeta_{\text{PC/MAE}}=\frac{\nu_{\text{MAE}}}{\nu_{\text{PC}}-\nu_{\text{MAE}}}$$

## Competing interests

The author(s) declare that they have no competing interests.

## Authors' contributions

Construction of the *pyk *deletion mutant was carried out by CK. JB performed the experimental studies of the present work, i.e. the physiological studies for determination of growth and production characteristics, the enzymatic analysis and the ^13^C tracer studies including GC/MS labeling analysis. CW carried out the modeling work including design and validation of the extended approach, metabolic modeling, parameter estimation and statistical analysis. The manuscript was drafted by CW and JB.
